# Supplementary material for: Pleiotrophin Interaction with Synthetic Glycosaminoglycan Mimetics
Source: Pharmaceuticals (Basel). 2022 Apr 19;15(5):496. doi: 10.3390/ph15050496 (PMC9147657; doi:10.3390/ph15050496)
Supplement: Supplementary file 1 [file pharmaceuticals-15-00496-s001.zip › pharmaceuticals-1628013-supplementary.pdf]

Pleiotrophin Interaction with Synthetic Glycosaminoglycan Mimetics

Jonathan R. Miles [1], Xu Wang [2], Jose L. de Paz [1] and Pedro M. Nieto [1,*]

[1] Glycosystems Laboratory, Instituto de Investigaciones Químicas (IIQ), cicCartuja, CSIC and Universidad de Sevilla, C/ Américo Vespucio, 49, 41092 Sevilla, Spain; jonathan.miles@nottingham.ac.uk (J.R.M.); jlpaz@iiq.csic.es (J.L.d.P.)

[2] School of Molecular Sciences, Arizona State University, Tempe, AZ 85281, USA; xuwang@asu.edu.

* Correspondence: pedro.nieto@iiq.csic.es

Table S1, ^{1}H and ^{15}N chemical shift of **1**

f2 (ppm)	f1 (ppm)	Intensidad	Anchura f2	Anchura f1	Volumen	Anotación
7.68	108.47	279822.5	21.91	32.24	87414107	81T
8.24	108.79	269329	19.49	42.77	99308687	110G
8.78	109.92	238231.5	18.25	35.64	68565033	75G
8.15	110.12	415844.5	16.74	35.64	1.1E+08	41G
8.42	110.43	422065.5	15.08	35.64	1E+08	64G
8.69	110.51	285509.5	17.12	35.64	77083033	33G
8.44	110.77	1348943	14.58	35.64	3.1E+08	128G
9.41	111.01	183648.5	20.26	35.64	58653982	31G
8.11	111.06	364049.5	19.62	33.27	1.05E+08	40T
7.72	111.29	516175	16.63	49.9	1.89E+08	102T
8.49	111.53	476892	15.86	35.64	1.19E+08	28G
9.09	112.49	186987.5	19.86	40.4	66372635	88G
8.46	112.66	236147	16.52	49.9	86092287	16G
8.05	113.12	295840.5	18.77	33.27	81736543	34T
8.01	114.03	507245	14.1	35.64	1.13E+08	30C
8.5	114.84	191665.5	20.17	42.77	73147241	38T
9.01	114.91	279166	19.94	35.64	87767779	85T
8.1	115.39	366691.5	15.26	38.02	94130341	22V
8.54	115.5	226759.5	18.61	42.77	79849801	96N
8.68	115.51	402186.5	16.28	35.64	1.03E+08	27S
8.24	115.9	249128.5	19.44	47.53	1.02E+08	89S
8.65	116.13	154809.5	18.15	35.64	44314403	47T
8.76	116.25	403107	15.89	40.4	1.14E+08	80N
8.7	116.37	226154.5	21.11	42.77	90319007	95H
8.15	116.52	1133919	14.96	35.64	2.67E+08	113T
9.09	116.8	334612	18.54	38.02	1.04E+08	43E
9.49	116.95	174861.5	22.52	33.27	57956422	37G
8.41	116.99	422353	19.62	47.53	1.74E+08	76E
8.7	117.19	373951	15.24	38.02	95842042	66E
8.42	117.21	386483.5	24.26	52.28	2.17E+08	50T
7.37	117.4	305916	19.84	35.64	95688834	83L
8.21	117.42	281779	19.98	40.4	1.01E+08	103V
8.38	117.74	1329173	19.05	33.27	3.73E+08	87T
8.36	117.81	919883	21.85	34.99	3.11E+08	104T
7.92	118.01	518407	15.42	35.64	1.26E+08	15C
8.24	118.22	362139.5	16.21	47.53	1.23E+08	109C
8.25	118.51	536021.5	15.3	64.16	2.33E+08	17E
8.57	118.51	265348	20	47.53	1.12E+08	106S
7.61	118.51	245171	18.61	35.64	71941136	92R
8.44	118.91	269426	21.51	47.53	1.22E+08	29D
7.73	119.06	338356	18.16	42.77	1.16E+08	67C

7.85	119.45	474899.5	17.23	40.4	1.46E+08	46Q
8.24	119.49	119741.5	20.31	83.17	89476018	39R
7.97	119.73	494085.5	16.13	35.65	1.26E+08	63F
8.19	120.02	321296.5	2.35	19.27	6431051	53C
7.45	120.04	506228.5	15.42	33.27	1.15E+08	44C
9.19	120.08	236086.5	17.93	42.77	80102944	21S
8.11	120.24	61771	14.19	36.48	14145403	74W
8.5	120.25	281983.5	19.25	52.28	1.26E+08	54K
9.12	120.45	195263	18.47	38.02	60658160	35R
8.16	120.47	819262	20.89	40.42	3.06E+08	62Q
8.49	120.69	446574	20.96	49.9	2.07E+08	98E
8.58	120.92	291881.5	35.55	40.4	1.85E+08	58N
8.59	120.99	285005.5	37.9	40.4	1.93E+08	70Q
8.45	121.04	1351089	12	35.64	2.56E+08	120E
7.86	121.41	343823	18.61	35.64	1.01E+08	48M
8.8	121.52	163905	21.59	47.9	74983279	57C
7.96	121.56	568177.5	15.09	33.27	1.26E+08	61K
8.24	121.56	1859902	18.63	52.16	8E+08	111K
8.29	121.56	209542.5	18.63	52.16	90074977	72Q
8.52	121.57	1317225	13.7	38.02	3.03E+08	118Q
8.01	121.68	511653.5	18.79	38.02	1.62E+08	59W
9.23	121.71	236361	15.71	38.02	62435026	26T
8.23	121.78	1811905	13.72	27.42	3.02E+08	2K
8.99	121.82	223849.5	16.35	35.64	57704838	77C
9.08	121.93	306882.5	29.16	42.77	1.69E+08	107K
9.07	122.14	321855	26.62	42.77	1.62E+08	84K
7.75	122.19	358239	17.31	35.64	97796626	60K
8.56	122.23	1560212	11.91	38.02	3.13E+08	7E
8.76	122.39	223896	36.1	40.34	1.44E+08	36E
8.3	122.5	430865.5	14.92	33.38	94921042	73A
7.79	122.54	542898.5	15.16	35.64	1.3E+08	97A
8.18	122.58	1644381	12.77	30.96	2.87E+08	126K
8.35	122.58	1634323	13.27	32.05	3.07E+08	8K
8.02	122.66	406895	17.86	38.02	1.22E+08	99C
8.47	122.7	1461111	2.35	11.88	18027951	23C
8.8	122.73	207780.5	30.36	44.31	1.24E+08	69Y
8.32	122.92	154061.5	16	29.99	32703660	18W
8.71	122.97	251594.5	12.13	44.42	59983542	82A
8.2	123.01	1701075	15.93	31.03	3.72E+08	10V
8.45	123.03	1281051	15.98	34.46	3.12E+08	134M
8.51	123.07	584569.5	13.48	43.08	1.5E+08	131Q
9.15	123.33	197659.5	22.71	40.4	80229165	19Q
8.52	123.41	1244277	13.92	36.83	2.82E+08	32L

8.5	123.54	3608129	13.92	36.83	8.18E+08	132E
8.87	123.59	335041	17.17	38.02	96745126	100Q
8.72	123.59	265834	22.24	45.74	1.2E+08	94L
8.56	123.71	553042	22.24	45.74	2.49E+08	49K
8.52	123.8	1367871	6.83	31.63	1.31E+08	9K
8.32	123.86	177425.5	17.81	33.89	47364416	112L
8.45	123.87	2550057	15.11	30.89	5.27E+08	116L
9.06	123.94	313045	26.44	40.4	1.48E+08	86R
9.05	123.96	327309.5	26.2	35.64	1.35E+08	51Q
8.42	124.11	2813103	15.52	29.45	5.69E+08	130K
8.88	124.3	225184	21.25	42.77	90531372	105I
8.29	124.57	1692201	13.87	31.48	3.27E+08	135L
8.53	124.77	358111.5	17.19	38.02	1.04E+08	52R
8.45	124.91	932906	13.34	35.64	1.96E+08	12K
9.07	125.11	306566.5	17.26	45.15	1.06E+08	101K
8.79	125.3	211244	17.18	40.4	64857427	20W
8.29	125.61	1107767	14.26	42.77	2.99E+08	114K
8.39	126.04	1390563	12.75	35.64	2.8E+08	119A
8.68	126.78	224511	25.85	49.9	1.28E+08	55I
8.44	126.92	1348230	12.73	33.27	2.53E+08	11K
7.89	126.95	1533245	13.32	33.27	3E+08	136D
8.68	127.17	198984	26.85	45.15	1.07E+08	45K
8.77	127.2	296440	15.62	40.4	82725276	79L
9.26	127.35	178929.5	18.91	35.64	53337459	71F
8.47	127.61	299136	17.65	35.64	83247302	93A
8.59	128.63	200507.5	16.42	38.02	55387618	90L
9.12	128.71	115825.5	23.4	45.15	54131436	91K
8.85	129.97	355753.5	14.6	33.27	76445835	42A
8.3	131.72	250226.5	17.09	35.64	67421578	78D
8.8	133.04	231257.5	19.4	35.64	70723821	24V

Table S2, ^{1}H and ^{15}N chemical shift of **2**

f2 (ppm)	f1 (ppm)	Intensidad	Anchura f2	Anchura f1	Volumen	Anotación
7.68	108.49	275943.3	19.59	33.26	79534491	81T
8.23	108.73	138776.4	19.49	42.77	51164174	110G
8.78	109.9	236697.5	18.25	35.64	68115075	75G
8.15	110.14	383922.1	16.74	35.64	1.01E+08	41G
8.68	110.48	333653.9	17.12	35.64	90070060	33G
8.43	110.5	516809	15.08	35.64	1.23E+08	64G
8.44	110.76	942095.9	14.58	35.64	2.17E+08	128G
9.39	111	191219.5	20.26	35.64	61064555	31G
8.1	111.06	330694.5	19.62	33.26	95489869	40T
7.71	111.32	491117.5	16.63	49.9	1.8E+08	102T
8.49	111.53	442972.6	15.86	35.64	1.11E+08	28G
9.09	112.49	205395.1	19.86	40.39	72897607	88G
8.46	112.67	228940.3	16.52	49.9	83454702	16G
8.06	113.16	320578.5	18.77	33.26	88560461	34T
8	113.97	560555.8	14.1	35.64	1.25E+08	30C
8.5	114.78	183310.1	20.17	42.77	69949817	38T
9.01	114.91	258019.7	19.94	35.64	81109479	85T
8.1	115.39	382389.9	15.26	38.02	98147937	22V
8.68	115.51	432116.6	16.28	35.64	1.11E+08	27S
8.51	115.63	123089.8	18.61	42.77	43338765	96N
8.23	115.87	273431.6	19.44	47.52	1.12E+08	89S
8.64	116.13	100689.2	18.15	35.64	28818851	47T
8.76	116.22	450247.5	15.89	40.39	1.28E+08	80N
8.68	116.31	158593.8	21.11	42.77	63329486	95H
8.15	116.58	813634.1	14.96	35.64	1.92E+08	113T
9.08	116.77	319742.8	18.54	38.02	99692684	43E
9.49	116.92	178456.7	22.52	33.26	59140781	37G
8.41	116.99	493966.5	19.62	47.52	2.04E+08	76E
8.42	117.21	361920.5	24.26	52.27	2.03E+08	50T
8.7	117.33	426163	15.24	38.02	1.09E+08	66E
7.37	117.4	310489.3	19.84	35.64	97107384	83L
8.2	117.43	317592.8	19.98	40.39	1.13E+08	103V
8.38	117.74	832645.6	19.05	33.26	2.33E+08	87T
8.36	117.81	578431.4	21.85	34.98	1.96E+08	104T
7.92	118.01	435311.5	15.42	35.64	1.06E+08	15C
8.25	118.17	658559.8	16.21	47.52	2.24E+08	109C
8.25	118.48	658559.8	15.3	64.15	2.86E+08	17E
8.57	118.49	276956.3	20	47.52	1.16E+08	106S
7.62	118.64	274880.5	18.61	35.64	80648820	92R
8.42	118.91	225439.9	21.51	47.52	1.02E+08	29D
8.25	119.01	522476.4	20.31	83.16	3.9E+08	39R

7.74	119.16	351076.8	18.16	42.77	1.21E+08	67C
7.84	119.49	334360	17.23	40.39	1.03E+08	46Q
7.98	119.82	480753.8	16.13	35.64	1.22E+08	63F
7.44	120.01	477897.4	15.42	33.26	1.08E+08	44C
9.19	120.08	232449.2	17.93	42.77	78859044	21S
8.18	120.35	16410.1	2.35	19.27	328423.6	53C
8.18	120.39	681300.4	20.89	40.42	2.54E+08	62Q
8.49	120.41	446002.6	20.96	49.9	2.06E+08	98E
8.14	120.43	426068.5	14.19	36.47	97556501	74T
9.12	120.45	224556.9	18.47	38.02	69749569	35R
8.48	120.59	505492.1	19.25	52.27	2.25E+08	54K
8.58	120.92	234180.3	35.55	40.39	1.49E+08	58N
8.59	120.99	238860.4	37.9	40.39	1.62E+08	70Q
8.45	121.04	1084336	12	35.64	2.05E+08	120E
7.85	121.38	278602.9	18.61	35.64	81737051	48M
8.79	121.49	85488.8	21.59	47.89	39104522	57C
8.29	121.57	213749.6	18.63	52.15	91872098	72Q
8.52	121.57	1023624	13.7	38.02	2.36E+08	118Q
7.96	121.61	541009.4	15.09	33.26	1.2E+08	61K
8.23	121.64	213749.6	18.63	52.15	91872098	111K
9.21	121.68	268073.5	15.71	38.02	70803254	26T
8.01	121.7	373871.2	18.79	38.02	1.18E+08	59W
8.99	121.82	136993.2	16.35	35.64	35310346	77C
8.22	121.87	8556.9	13.72	27.42	1423810	2K
9.07	122.05	320165.8	26.62	42.77	1.61E+08	84K
9.07	122.09	319398.9	29.16	42.77	1.76E+08	107K
8.44	122.19	7332.9	2.35	11.88	90466.49	23C
7.75	122.22	307329.2	17.31	35.64	83888401	60K
8.56	122.29	1507032	11.91	38.02	3.02E+08	7E
8.76	122.39	215530.6	36.1	40.34	1.39E+08	36E
8.3	122.5	447429.2	14.92	33.37	98557999	73A
8.17	122.52	1203480	12.77	30.96	2.1E+08	126K
7.79	122.53	501804.8	15.16	35.64	1.2E+08	97A
8.35	122.58	1563903	13.27	32.04	2.94E+08	8K
8	122.59	406640.8	17.86	38.02	1.22E+08	99C
8.79	122.66	236988.7	30.36	44.3	1.41E+08	69Y
8.47	122.66	976747.2	13.48	43.07	2.51E+08	131Q
8.32	122.92	169616.1	16	29.99	36001109	18W
8.71	122.93	248074.9	12.13	44.41	59137239	82A
8.21	122.95	1659390	15.93	31.03	3.63E+08	10V
8.44	123.01	1065065	15.98	34.46	2.59E+08	134M
8.5	123.24	1292622	13.92	36.83	2.93E+08	132E
9.15	123.33	197156.5	22.71	40.39	80015054	19Q

8.69	123.4	224379.4	24	74.44	1.77E+08	68C
8.69	123.4	418315.2	22.24	45.73	1.88E+08	94L
8.5	123.52	2492035	13.92	36.83	5.65E+08	32L
8.87	123.6	322527.1	17.17	38.02	93120115	100Q
8.52	123.7	91013.1	6.83	31.63	8692600	9K
8.56	123.71	406481.4	22.24	45.73	1.83E+08	49K
8.45	123.87	2220212	15.11	30.89	4.58E+08	116L
9.06	123.93	330193.6	26.44	40.39	1.56E+08	86R
9.05	123.96	328331.1	26.2	35.64	1.36E+08	51Q
8.34	124.06	258139.8	17.81	33.88	68902903	112L
8.42	124.11	2129506	15.52	29.45	4.31E+08	130K
8.88	124.38	237987.4	21.25	42.77	95667039	105I
8.29	124.53	2030897	13.87	31.47	3.92E+08	135L
8.53	124.73	396633.9	17.19	38.02	1.15E+08	52R
8.45	124.9	858863	13.34	35.64	1.81E+08	12K
9.07	125.11	255879.3	17.26	45.14	88181403	101K
8.79	125.3	243894.6	17.18	40.39	74872722	20W
8.29	125.61	898189.9	14.26	42.77	2.42E+08	114K
8.39	126.04	969674.1	12.75	35.64	1.95E+08	119A
8.68	126.87	239093.2	26.85	45.14	1.28E+08	45K
8.68	126.91	234392.4	25.85	49.9	1.34E+08	55I
8.44	126.92	1281726	12.73	33.26	2.4E+08	11K
7.89	126.95	2708321	13.32	33.26	5.31E+08	136D
8.77	127.2	347446.3	15.62	40.39	96947231	79L
9.26	127.35	166213.1	18.91	35.64	49540628	71F
8.47	127.61	389342.8	17.65	35.64	1.08E+08	93A
8.58	128.6	217615.7	16.42	38.02	60106109	90L
9.1	128.69	136476.9	23.4	45.14	63775079	91K
8.85	129.97	347570	14.6	33.26	74678221	42A
8.29	131.7	246073.2	17.09	35.64	66294423	78D
8.8	133.04	241974	19.4	35.64	73991999	24V

Table S3, ^{1}H and ^{15}N Kd and CSP(max) of **1**

Pico	Kd	σ(Kd)	CSP Máx	σ(CSP Máx)
2K	0.20467	0.08425	0.01916	0.00199
7E	0.25322	0.26844	0.01609	0.00464
8K	0.00722	0.01927	0.0013	0.00044
9K	0.30337	0.09766	0.00921	0.00091
10V	0.42371	0.07111	0.04339	0.00239
11K	1.32E-09	0.0324	0.01411	0.00122
12K	0.01942	0.16085	0.00966	0.00368
7E	0.25322	0.26844	0.01609	0.00464
8K	0.00722	0.01927	0.0013	0.00044
9K	0.30337	0.09766	0.00921	0.00091
10V	0.42371	0.07111	0.04339	0.00239
11K	1.32E-09	0.0324	0.01411	0.00122
12K	0.01942	0.16085	0.00966	0.00368
15C	0.04105	0.09059	0.01908	0.00371
16G	0.18212	0.17502	0.00687	0.00098
17E	0.51413	0.12734	0.22335	0.02201
18W	0.28079	0.02349	0.05711	0.00151
19Q	0.15996	541.082	0.00533	3.52391
20W	0.8269	13.2483	0.01784	0.13365
21S	0.00845	0.00451	0.01294	0.00039
22V	0.06784	0.26799	0.02272	0.00982
23C	0.32761	0.04118	0.01783	0.00067
24V	2.89E-10	0.05701	0.00578	0.00066
26T	0.16115	0.1165	0.02222	0.0035
27S	0.02167	0.00694	0.01413	0.00043
28G	0.09475	0.08598	0.03152	0.00445
29D	0.35515	0.08233	0.03282	0.002
30C	0.0042	0.00379	0.00173	9.28E-05
31G	0.06398	0.08366	0.05376	0.00827
32L	0.03275	0.01531	0.00802	0.00027
33G	0.05281	0.02726	0.02039	0.00114
34T	0.06392	0.01253	0.02246	0.00037
35R	0.04146	0.01936	0.00755	0.00034
36E	3.17E-10	0.22999	0.04094	0.01535
37G	1.00926	388.625	0.04303	8.81105
38T	1.0833	3.14811	0.01721	0.02729
39R	0.50494	0.40427	0.02207	0.00695
40T	2.39256	762.636	0.03944	9.42097
41G	0.01191	0.00994	0.00254	0.00043
42A	1.30756	0.47712	0.01369	0.00291
43E	0.02547	0.01159	0.00669	0.00013

44C	0.36362	0.0692	0.00252	0.00015
45K	0.29663	0.02583	0.06368	0.0014
46Q	0.56155	0.72072	0.0555	0.0289
47T	0.71826	0.30896	0.03749	0.0075
48M	0.20665	0.11026	0.00751	0.00102
49L	0.00039	0.00209	0.00346	9.38E-05
50T	0.3003	0.05069	0.03169	0.00148
51Q	0.41325	0.09758	0.02292	0.0019
52R	0.42531	0.15792	0.10344	0.01411
53C	0.06414	0.01506	0.05995	0.00112
54K	0.21075	0.04268	0.09255	0.00396
55L	1.32319	0.15944	0.08894	0.00513
57C	0.15673	0.08153	0.14177	0.01602
58N	0.34676	0.03263	0.00245	7.00E-05
59W	0.12637	0.02261	0.1248	0.00301
60K	0.14705	0.14022	0.16384	0.03112
61K	0.11665	0.02427	0.10669	0.00486
62Q	0.38371	0.45193	0.28694	0.11378
63F	0.123	0.05838	0.29323	0.02647
64G	0.1748	0.0752	0.25906	0.02594
66E	0.05994	0.04293	0.17579	0.01536
67C	0.08104	0.08144	0.19371	0.02647
68C	0.34676	0.03263	0.04215	0.0012
68C	0.40029	0.72046	0.02453	0.01494
69Y	0.07124	0.0477	0.10795	0.00954
70Q	0.01831	0.00451	0.04204	0.00054
71F	0.01032	0.00918	0.0162	0.00095
72Q	1.53671	0.14905	0.08055	0.00449
73A	0.00228	0.00405	0.00417	0.00146
74T	0.18019	0.06827	0.02971	0.00187
75G	0.26785	0.18831	0.03285	0.00648
76E	0.37011	0.09329	0.02808	0.0026
77C	0.01174	0.00641	0.02687	0.00087
78D	2.87194	218.402	0.02177	1.29995
79L	0.06541	0.01784	0.00709	0.00026
80N	3.5404	756.285	0.0513	9.27781
81T	0.03056	0.02606	0.00756	0.00076
82A	0.00375	0.00625	0.00191	7.34E-05
83L	0.47448	0.12152	0.01678	0.0017
84K	0.0122	0.00691	0.01835	0.00087
85T	0.13043	0.19965	0.02959	0.00798
86R	2.30356	0.38672	0.09946	0.01118
87T	0.43107	0.71807	0.03295	0.01934

88G	0.07659	0.08152	0.05547	0.0078
89S	4.94E-10	0.04216	0.00587	0.00063
90L	0.08	0.05485	0.0251	0.00302
91K	0.134	0.03202	0.14328	0.00623
92R	0.23609	0.08613	0.20379	0.02082
93A	0.15523	0.08123	0.01424	0.0015
94L	0.1105	0.08056	0.21191	0.02628
95H	0.39805	0.08057	0.11525	0.00915
96N	0.08787	0.08433	0.17308	0.02393
97A	0.07722	0.06996	0.05687	0.00687
98E	0.34676	0.03263	0.04545	0.0013
99C	0.12413	0.16112	0.15118	0.03396
100Q	0.12069	0.03953	0.02532	0.00164
101K	0.34676	0.03263	0.00097	2.78E-05
102T	8.87E-10	0.15169	0.02962	0.00794
103V	0.00597	0.00577	0.02015	0.00095
104T	0.52406	0.05348	0.06589	0.00218
105I	0.22348	0.02425	0.07065	0.00148
106S	0.17193	0.09011	0.02162	0.00302
107K	0.0122	0.00691	0.01835	0.00087
109C	0.40764	0.0623	0.03338	0.00183
110G	0.00348	0.00629	0.00476	0.00045
111K	0.07075	0.2074	0.00928	0.00316
112L	0.075	0.02009	0.03828	0.00138
113T	0.20035	0.23116	0.04999	0.01348
114K	0.03744	0.01898	0.02557	0.00123
116L	0.14455	0.01652	0.00939	0.00018
118Q	0.44548	1.64374	0.02865	0.03692
119A	0.04862	0.14126	0.01723	0.00469
120E	0.27418	0.26887	0.02903	0.00815
126K	0.44837	1.04279	0.02349	0.01935
128G	0.00398	0.00501	0.00626	0.00078
130K	0.40981	2.27387	0.02352	0.04299
131Q	0.06973	0.03509	0.00791	0.00036
132E	0.66643	0.08779	0.0456	0.00254
134M	0.34676	0.03263	0.00128	3.64E-05
135L	1.07249	504.382	0.01281	3.25286
136D	0.47497	0.20641	0.00229	0.00031

Table S4, ^{1}H and ^{15}N Kd and CSP(max) of **2**

Pico	Kd	σ(Kd)	CSP Máx	σ(CSP Máx)
2K	0.02233	0.02121	0.02365	0.00161
7E	1.18405	0.11014	0.06784	0.00336
8K	1.80078	0.49568	0.06083	0.00883
9K	0.44356	0.0516	0.03606	0.00157
10V	2.37307	0.20515	0.1357	0.00733
11K	3.56651	0.54603	0.1777	0.01809
12K	0.94536	0.18352	0.06067	0.00481
15C	11.9647	3.08199	0.33892	0.07752
16G	2.43441	1.82711	0.06752	0.03401
17E	0.26593	0.03105	0.09295	0.00297
19Q	0.32414	0.13423	0.02607	0.00294
20W	0.17269	0.05111	0.01465	0.00095
21S	11.2679	1.88771	0.34993	0.04987
22V	1.9235	0.15275	0.12969	0.00619
23C	0.70875	0.47462	0.04163	0.01189
24V	0.33726	0.03813	0.03443	0.00093
27S	0.72921	0.10569	0.05518	0.00318
28G	1.35721	0.50733	0.11349	0.02373
29D	5.35483	5.10678	0.1534	0.11679
30C	5.04842	304.537	0.14904	7.18447
31G	0.15133	0.02796	0.06042	0.00154
32L	0.0496	0.00627	0.02614	0.00033
33G	0.22885	0.10552	0.01807	0.00132
34T	3.0316	0.47366	0.20149	0.02001
35R	4.13184	1.49922	0.10096	0.02704
36E	0.00986	0.01947	0.04943	0.00218
37G	2.19244	0.27531	0.13996	0.01002
38T	8.32103	3.82729	0.25318	0.09463
39R	0.3963	0.05161	0.03281	0.0012
40T	7.92759	12.5807	0.23448	0.31659
41G	1.45411	0.55665	0.04219	0.00918
42A	2.46631	0.53312	0.08146	0.01175
43E	2.95621	0.52449	0.08946	0.00962
44C	0.13727	0.08996	0.01439	0.00184
45K	0.03187	0.01194	0.02078	0.00086
46Q	121.134	409.881	6.0246	20.1307
47T	0.40242	0.02709	0.10316	0.00221
48M	16.6139	13.6336	0.49297	0.35486
49K	0.40242	0.02709	0.03557	0.00076
50T	0.40242	0.02709	0.01237	0.00027
51Q	2.50215	1.13609	0.08954	0.02553

52R	0.32843	0.03379	0.06594	0.00193
53C	0.53505	0.1257	0.2416	0.02502
54K	0.4058	0.03242	0.02349	0.00055
55L	2.68293	0.28242	0.29461	0.01881
57C	1.23741	0.27864	0.37495	0.04433
58N	0.0258	0.00832	0.03058	0.00045
59W	0.20829	0.01137	0.27378	0.00285
60K	1.00564	0.11435	0.45474	0.02076
61K	0.22463	0.04224	0.07507	0.00406
62Q	0.78816	0.12528	0.54314	0.03736
63F	0.41114	0.07878	0.4489	0.02823
64G	1.20809	0.1793	0.63729	0.05106
66E	0.48421	0.06598	0.28608	0.01386
67C	0.6756	0.11023	0.36693	0.02293
69Y	0.22224	0.02483	0.11636	0.00352
70Q	0.47398	0.05614	0.02378	0.001
71F	2.44572	0.70975	0.0659	0.01215
72Q	0.06827	0.01005	0.04214	0.00065
73A	0.02437	0.06331	0.01197	0.00233
74W	0.00064	0.00217	0.02894	0.00385
75G	1.28775	0.15016	0.08311	0.0043
76E	0.40242	0.02709	0.05614	0.0012
77C	0.87366	0.19733	0.05715	0.00613
78D	1.41561	0.63353	0.04685	0.01189
79L	0.65283	0.07223	0.03064	0.00125
80N	0.40242	0.02709	0.01156	0.00025
81T	0.30577	213.924	0.00023	0.04668
82A	0.53901	0.2281	0.02509	0.00345
83L	1.30091	0.06497	0.06052	0.00153
84K	0.27776	0.03659	0.03605	0.0014
85T	1.1417	0.14729	0.06956	0.00484
86R	0.31144	0.07614	0.06347	0.00495
87T	3.45093	0.60149	0.18496	0.02255
88G	0.78605	0.07535	0.10042	0.0036
89S	0.38218	0.04196	0.02738	0.00082
90L	0.52487	22.6321	0.00733	0.0736
91K	0.61141	0.10077	0.17419	0.01076
92R	0.51767	0.04277	0.29482	0.00756
93A	0.90513	528.729	0.0135	2.69124
94L	0.08555	0.03951	0.13192	0.00894
95H	0.08008	0.00883	0.06773	0.00118
96N	0.55409	0.0444	0.25417	0.00639
97A	1.53617	0.15597	0.18554	0.0088

98E	5.90863	212.528	0.45859	13.5702
99C	0.96011	0.18563	0.24556	0.01802
100Q	1.02041	0.20055	0.04214	0.0038
101K	0.51261	0.24006	0.02293	0.00307
102T	0.00058	0.00289	0.01387	0.00037
103V	0.81948	0.23474	0.0466	0.00571
104T	0.33087	0.03012	0.08097	0.0024
105I	0.40242	0.02709	0.08997	0.00193
106S	2.03648	0.44889	0.08092	0.01093
107K	0.29309	0.01478	0.04409	0.00057
109C	0.12461	0.01485	0.04094	0.001
110G	2.24871	0.72543	0.08452	0.01747
111K	3.26406	0.55799	0.10479	0.01282
112L	0.79508	0.07061	0.09322	0.00352
113T	2.1663	0.21976	0.21388	0.01179
114K	2.10004	0.1493	0.14514	0.0062
116L	4.23851	0.57158	0.14365	0.01473
118Q	1.98391	0.25817	0.10844	0.00786
119A	2.217	0.29677	0.12285	0.00911
120E	1.53343	0.22281	0.08469	0.00579
126K	4.41405	0.60555	0.17751	0.01806
128G	5.22809	1.13581	0.1556	0.02426
130K	4.82849	0.78115	0.1837	0.02101
131Q	0.40242	0.02709	0.03277	0.0007
132E	0.94804	0.18023	0.04755	0.00404
134M	0.10292	0.01689	0.01922	0.00043
135L	0.47561	0.05689	0.04528	0.00162
136D	0.71721	0.06723	0.03	0.00097
